# Data on the localization of EGFP and 20α-hydroxysteroid dehydrogenase (20α-HSD) in the placenta and testes of transgenic mice

**DOI:** 10.1016/j.dib.2018.05.069

**Published:** 2018-05-19

**Authors:** Mi-Hyang Yoo, Chae-Won Park, Munkhzaya Byambaragchaa, Myung-Hwa Kang, Kwan-Sik Min

**Affiliations:** a Animal Biotechnology, Graduate School of Future Convergence Technology, Institute of Genetic Engineering, Dept. of Animal Life Science, Hankyong National University, Anseong 17579, Republic of Korea; b Department of Food Science & Nutrition, Hoseo University, Asan 31499, Republic of Korea

**Keywords:** Transgenic mice, 20α-HSD promoter, Testis, Placenta

## Abstract

In order to investigate the function of monkey 20α-hydroxysteroid dehydrogenase (20α-HSD), transgenic mice (tg) were produced, expressing enhanced green fluorescent protein (EGFP) under the control of the monkey 20α-HSD promoter. The expression levels and localization of EGFP and 20α-HSD were analyzed in immature testis and in placenta. In support of our recent publication, "Characterization of transgenic mice expressing EGFP under control of monkey 20α-hydroxysteroid dehydrogenase promoter" (Park et al., 2018) [1], it was important to characterize the function of EGFP and 20α-HSD in the ovarian luteal cells of tg mice. Here, the expression of EGFP and 20α-HSD in immature testis and placenta are presented. The expression level of EGFP and 20α-HSD were detected in the testes 1 week after birth, and increased dramatically at 8 weeks. Both of proteins strongly detected in the placenta on days 14, 16, and 18 of pregnancy. Immunohistochemical analysis revealed that EGFP was detected in the seminiferous epithelium and 20α-HSD was specifically localized in the seminiferous tubule at 8 weeks.

**Specifications Table**

**Table:** 

Subject area	*Biology*
More specific	*Monkey 20a-HSD promoter*
subject area	
Type of data	*Figures, graphs, and Western blots*
How data was	*RT-PCR, qRT-PCR, Western blotting and immunohistochemistry* acquired
Data format	*Analyzed*
Experimental	*Expression analyses of EGFP and 20a-HSD in immature testes and* factors *placenta of tg mice*
Experimental	*Characterization of EGFP and 20a-HSD using RT-PCR and qRT-* features *PCR, western blotting and confocal microscopy to determine the localization in the testes and placenta*
Data source	*Anseong, Korea*
location	
Data accessibility	*Data presented in this article*

**Value of the data**

• IHC analysis suggests that endogenous 20α-HSD can serve as a key role in the testis seminiferous and in chorionic- and trophoblast villus of the placenta.
• 20α-HSD localization in immature testes and placenta suggest a potential role in immature testes after birth and in the placenta during pregnancy.
• Testes and placenta substantially contribute to steroid hormone regulation during pregnancy and after birth.

## 1 Data

**Fig. 1:**
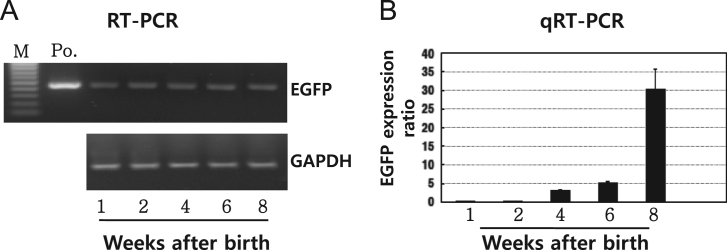
mRNA expression of EGFP in mice. Total RNA was subjected to RT-PCR (A) and qRT-PCR (B). The amplified products of *EGFP* and glyceraldehyde 3-phosphate (*GAPDH*) genes were separated on an agarose gel. qRT-PCR was performed using LightCycler® 96 and a FastStart Essential DNA Green Master Kit. The results were analyzed by comparing the quantification cycle (C_q_) value of 20α-HSD to that of *GAPDH*. M: marker; PC: positive control.

**Fig. 2:**
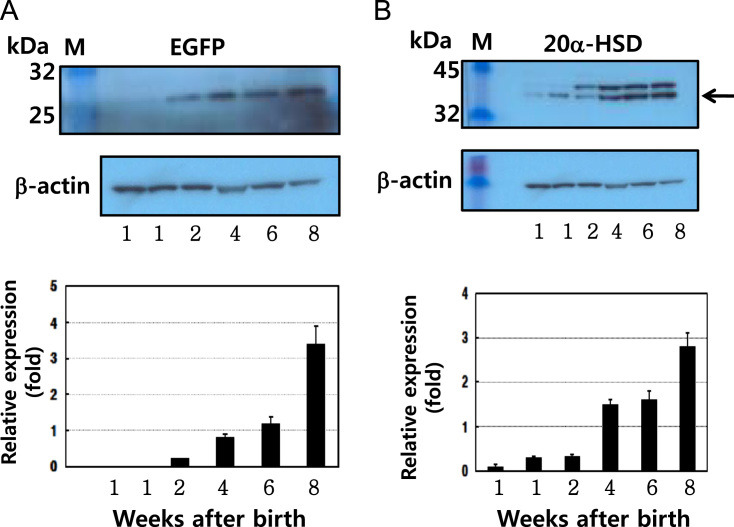
Protein expression of EGFP and 20α-HSD in mice testes. Testes were obtained from 12 mice. The extracted proteins were bound with primary antibodies, followed by anti-mouse/rabbit IgG-POD secondary antibodies; β-actin was used as the loading control. We detected 27- and 37-kDa bands on the membranes (EGFP and 20α-HSD, respectively). The relative expression levels of EGFP and 20α-HSD present in three sets of samples evaluated by western blotting (right).

**Fig. 3:**
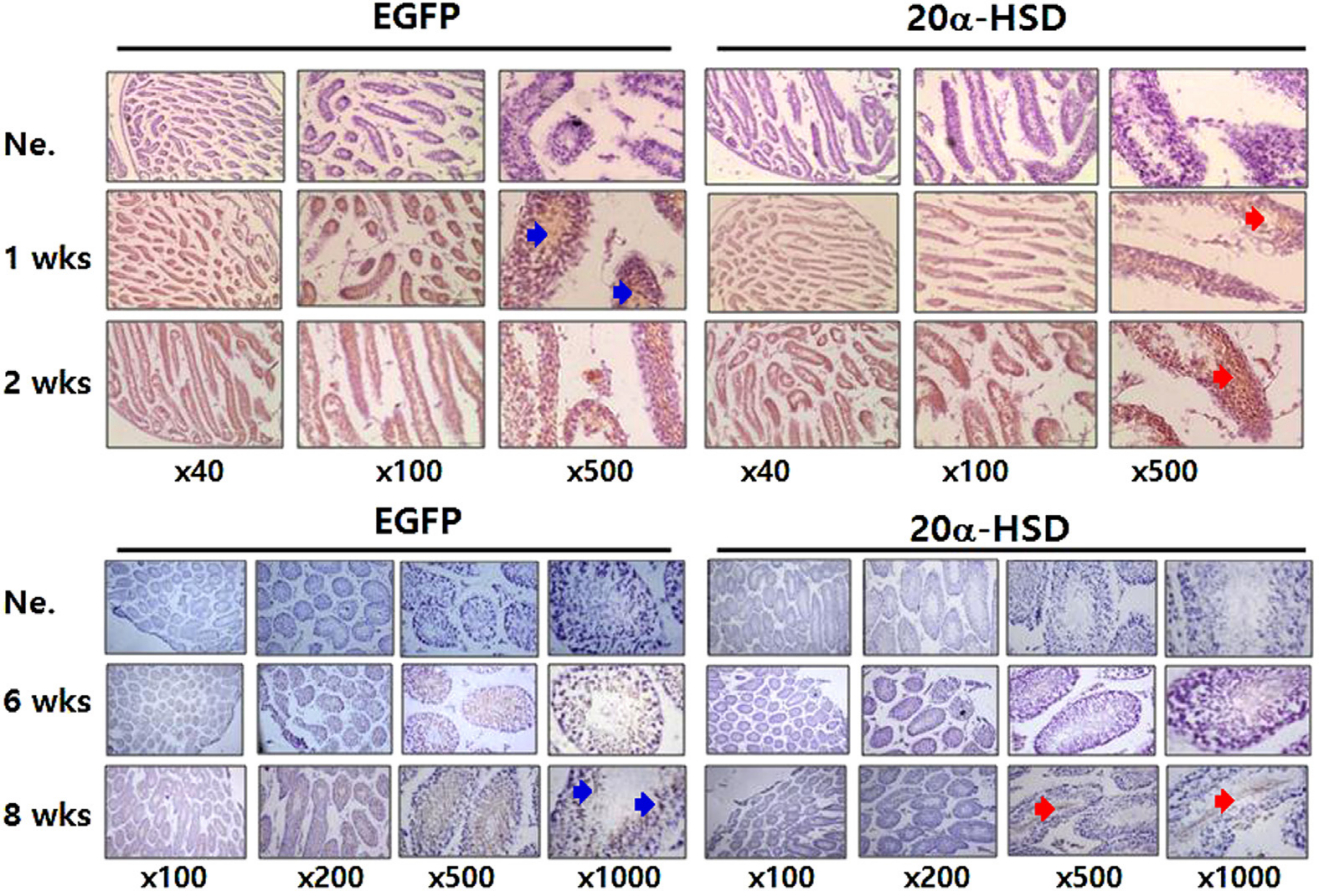
Localization of EGFP and 20α-HSD in mice testes. Representative immunohistochemical (IHC) analyses using EGFP and 20α-HSD antiserum. Proteins were detected with anti-EGFP (1:2500) and anti-20α-HSD (1:1000) antibodies. IHC analyses were performed using a Vectastain ABC kit. Pre-immune serum was used as the control for testes staining. EGFP protein was mainly localized in the seminiferous. 20α-HSD expression was specifically localized in some seminiferous tubules.

**Fig. 4:**
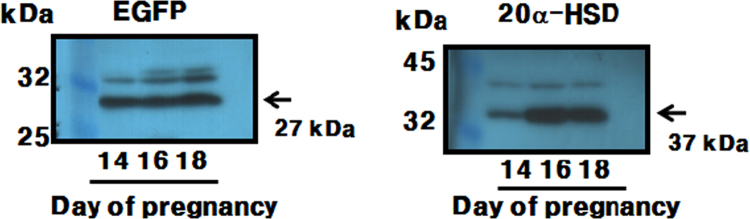
Protein expression of EGFP and 20α-HSD in mice placenta during pregnancy. Placentas were obtained on days 14, 16, and 18 of pregnancy. Proteins extracted from the placentas were transferred to PVDF membranes and used anti-EGFP (left) and anti-20α-HSD (right) primary antibodies, followed by anti-mouse/rabbit IgG-POD secondary antibodies. The proteins of EGFP and 20α-HSD were revealed a strong, 27- and 37-kDa molecular weight, respectively.

**Fig. 5:**
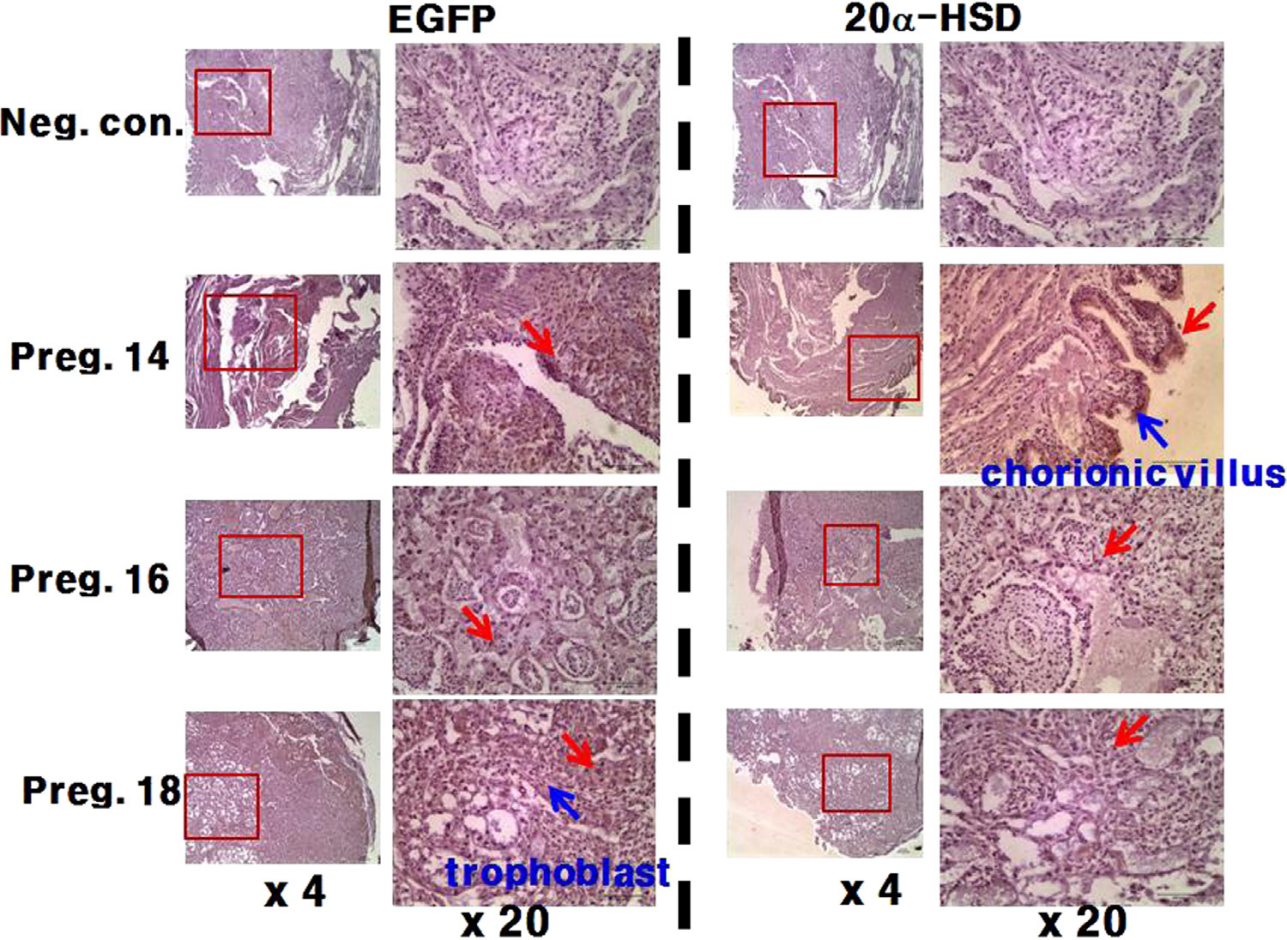
Localization of EGFP and 20α-HSD in mice placenta on days 14, 16, and 18 of pregnancy. For the detection of proteins the primary antibody used anti-EGFP (left) and anti-20α-HSD (right) antibodies. IHC analyses were performed using a Vectastain ABC kit. Negative control: staining with pre-immune serum. The specific signal was localized to the trophoblast and chorionic villi.

EGFP mRNA was detected and analyzed in the tg mice testes after birth using the specific primers for EGFP (Fig. 1A,B). EGFP and 20α-HSD protein expression in the tg mice testes 1, 2, 4, 6, and 8 weeks after birth were detected by western blotting (Fig. 2A,B). The specific antibody in our lab was used for examining the localization of EGFP and 20α-HSD protein in the immature testes (Fig. 3). Western blot (Fig. 4) and immunohistochemistry (Fig. 5) results of both protein in the placenta during pregnancy were described.

## 2 Experimental design, materials and methods

### 2.1 Experimental design and samples from transgenic mice

Monkey 20α-HSD promoter was cloned into the pCR2.1 vector. The PCR products of EGFP+bGHpolyA were disgested with *Eco*RV and *Xho*I and then ligated into the vector under the monkey 20α-HSD promoter. The transgene fragments were released from the vector by restriction enzyme digestions (*Eco*RI and *Xho*I) and microinjected into fertilized eggs to produce tg mice.

We bred tg mice expressing the EGFP gene under the control of the monkey 20α-HSD gene promoter [1]. C57BL/6N mice were used in the experiments. Tg mice were obtained by crossing PCR-positive founder (F0) mice with their wild-type (control) littermates. The positive male/female mice were crossed to increase the prevalence of homozygous mutant mice. The testes samples were collected on days 14, 16, and 18 during pregnancy. Samples were also obtained from the testes 1, 2, 4, 6, and 8 weeks after parturition. Animal housing and all animal experiments were performed in accordance with the Hankyong National University Animal Care and Use Committee Guideline (Approval No. HKNU 2016-01).

### 2.2 Reverse transcription polymerase chain reaction (RT-PCR)and qRT-PCR

The synthesized cDNA was used in each RT-PCR. The EGFP gene was amplified using a forward primer (5′-ACA AGC AGA CAG TGT GTC CAG GGG-3′) and a reverse primer (5′-TGT AGT TGC CGT CGT CCT TGA AGA-3′). The PCR conditions were: 1 cycle of pre-denaturation at 95 °C for 5 min; 30 cycles of denaturation at 94 °C for 40 s, annealing at 64 °C for 40 s, extension at 72 °C for 50 s; 1 final cycle of extension at 72 °C for 10 min; and cooling to 4 °C. Primers for the glyceraldehyde 3-phosphate gene (*GAPDH*) were used for normalization of EGFP expression, and the primer sequences were forward primer (5′-ACC ACA GTC CAT GCC ATC AC-3′); and reverse primer (5′-TCC ACC ACC CTG TTG CTG TA-3′) [2]. The PCR product (10 uL) was analyzed using gel electrophoresis, and the expected size was 400 bp. EGFP mRNA was expressed 1 week after birth in the testes of the tg mice. EGFP mRNA was detected in all the testes samples 1–8 weeks after birth (Fig. 1A). We then performed qRT-PCR to determine the level of EGFP mRNA expression (Fig. 1B).

### 2.3 EGFP and 20α-HSD protein expression in the tg mice testes

Total proteins were extracted from 10–20 mg of the tissue (testis), using a Pro-Prep™ protein extraction solution. The proteins were transferred to polyvinylidene difluoride (PVDF) (0.2 μm) using a semidry electroblotter apparatus. The membranes were incubated overnight at 4 °C with the primary antibody (1:5000) (20α-HSD antibody raised in our lab) diluted within in 1% blocking buffer, washed to remove unbound antibody, and incubated with an anti-rabbit IgG-H&L secondary antibody (1:3000) for 2 h [3]. Subsequently, the membranes were incubated with Lumi-Light substrate solution and exposed to X-rays for 1–10 min. Western blotting of the proteins extracted from the testes 1, 2, 4, and 6, and 8 weeks after birth (Fig. 2A,B) revealed the expected EGFP band (27 kDa) and 20α-HSD band (37 kDa). EGFP protein was not detected after 1 week in the testes. However, it was weakly expressed at 2 weeks, and protein expression increased slightly at 4 and 6 weeks. The highest expression of the protein was at 8 weeks after birth.

### 2.4 Immunohistochemistry of tg mice testes

Immunohistochemical (IHC) staining of tg mice testes samples (1, 2, 6, and 8 weeks after birth) was performed using the Vectastain ABC kit according to the method described previously [4]. The sections were incubated overnight at 4 °C with the primary antibody (1:500) diluted in 5% horse serum blocking buffer, and then with a biotinylated secondary antibody (1:1000). Tissue sections were immunostained using the ABC detection kit and stained with DAB (Fig. 3)**.** EGFP protein was mainly localized in the seminiferous epithelium of the tg mice testes at 1 to 8 weeks. 20α-HSD had similar localization to EGFP at 1, 2, and 6 weeks. However, 20α-HSD expression was specifically localized in some seminiferous tubules.

### 2.5 Western blotting analysis in the tg mice placenta

Total proteins were extracted from 10–20 mg of the tissue (placenta), using a Pro-Prep™ protein extraction solution. After transfer, the membranes were incubated overnight at 4 °C with the primary antibody (1:5000) (20α-HSD antibody raised in our lab) diluted within in 1% blocking buffer, and incubated with an anti-rabbit IgG-H&L secondary antibody (1:3000) for 2 h. Western blotting revealed a strong, 27-kDa band corresponding to EGFP in the placental tissue obtained from tg mice on days 14, 16, and 18 of pregnancy (Fig. 4).

### 2.6 Immunohistochemistry of tg mice placenta

We carried out IHC analysis of tissues on days 14, 16, and 18 of pregnancy to identify the types of cell in the placenta of tg mice that expressed EGFP. EGFP expression was mainly localized in the chorionic villi and the trophoblastic cells of the placenta (Fig. 5). We also determined that mouse 20α-HSD was localized at the same sites: Fig. 5 indicates that mouse 20α-HSD was strongly expressed in the chorionic villi of the placenta.
