# Supplementary figures and images for: Data on the localization of EGFP and 20α-hydroxysteroid dehydrogenase (20α-HSD) in the placenta and testes of transgenic mice
Source: Data Brief. 2018 May 19;19:632–7. doi: 10.1016/j.dib.2018.05.069 (PMC5997899; doi:10.1016/j.dib.2018.05.069)

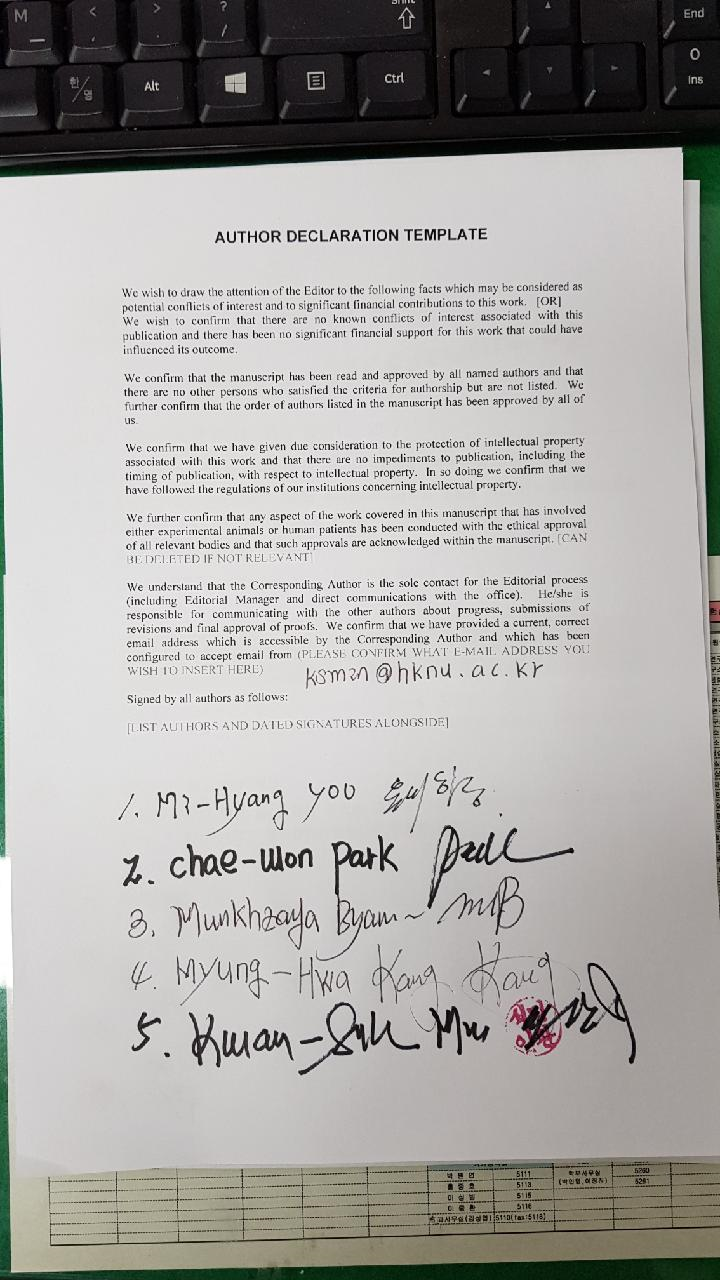

Supplement: Supplementary file 1 — Supplementary material [file mmc1.docx]
